# The health equity characteristics of research exploring the unmet community mobility needs of older adults: a scoping review

**DOI:** 10.1186/s12877-022-03492-8

**Published:** 2022-10-20

**Authors:** Hester van Biljon, Lana van Niekerk, Isabel Margot-Cattin, Fasloen Adams, Nicola Plastow, David Bellagamba, Anders Kottorp, Ann-Helen Patomella

**Affiliations:** 1 Stellenbosch University, Stellenbosch, South Africa; 2 University of Applied Sciences and Arts, Windisch, Switzerland; 3 Malmö University, Malmö, Sweden; 4 Karolinska Institutet, Solna, Sweden

**Keywords:** Active ageing, Research equity, Vulnerable groups, Inconsistent reporting, Sustainable development, Knowledge gap, Minority groups, Discrimination, Exclusion

## Abstract

**Background:**

Unmet community mobility needs of older adults, published since the announcement of the UN sustainable development goals was synthesised to describe the health equity characteristics of research identifying unmet community mobility needs of older adults.

**Methods:**

Searches were conducted in March and April 2020, 2275 articles were screened and 100 identified for data extraction.

**Results:**

Findings showed underrepresentation of articles considering rural settings [9%] and originating in the global South [14%]. Gender, disability, education, and transport / driving were identified as key health equity characteristics and only 10 articles provided detail on all four of these. External factors inhibiting community mobility included built environments, service availability, and societal attitudes. Internal factors included finances, fear and apprehension, and functional limitations.

**Conclusions:**

The need for standardised reporting of participant characteristics in the community mobility of older adults was highlighted. These characteristics are required by research consumers to judge equity dimensions, and the extent to which findings represent minority or marginalised groups. 15 after the UN pledge to reduce inequalities, peer reviewed primary research does not reflect a global drive to end discrimination, exclusion and reduce the inequalities and vulnerabilities that leave people behind.

**Supplementary Information:**

The online version contains supplementary material available at 10.1186/s12877-022-03492-8.

## Background

In September 2015, the United Nations (UN) promulgated the 2030 Agenda for Sustainable Development plan [1] with the pledge to eradicate poverty in all its forms, end discrimination and exclusion, and reduce the inequalities and vulnerabilities that leave people behind and undermine the potential of individuals [1]. This pledge holds special relevance to older adults, who, as a vulnerable population group, could find themselves discriminated against, shunned, and excluded from participating in societies they identify with, partly due to unmet community mobility needs [2, 3]. Furthermore, during times of social and environmental disasters [4], or pandemics, such as the COVID-19 pandemic and its associated regulations and disruptions [5], older adult population are at risk of being further marginalised. This scoping review was conducted midst the global Coronavirus disease (COVID-19) pandemic.

The UN’s Convention on the Rights of Persons with Disabilities (CRPD) [6] applies to older adults, since the first article within the CRPD includes those who have “…long-term physical, mental, intellectual or sensory impairments which in interaction with various barriers may hinder their full and effective participation in society on an equal basis with others”. As such, age-friendly communities aim at recognizing the older adult as an actor in society, and making them feel secure, understood, respected, and supported [7]. Community mobility is critical for older people to participate in their chosen occupations and be actors within society fully and effectively.

Community mobility comprises moving about in life space outside one's home [8]. The term’life space’, as used in gerontology, relates to mobility and navigating outside one’s home to reach the places where participation in the community unfolds [9] The construct of community as defined by an individual, implies freedom of association and locational choices [10]. Thus, community mobility comprises navigating of life space in order to reach places that are meaningful, fostering a sense of belonging, and supporting social participation [11] by engaging in occupations that are of value to the person [12]. Community mobility is a key determinant of health and quality of life [13] that can be affected by a wide variety of factors ranging from personal, internal factors to external and global factors. Navigating safely in the community is crucial for social participation, physical and mental health [14] and is dependent on older adult’s community mobility needs being fulfilled [15].

Although no comprehensive framework or consensual definition for community mobility needs were found in the literature, various types of needs were identified, as well as problems related to unmet needs [11]. Firstly, transportation needs to be accessible [16] available [17], affordable [18], and safe [19]. Secondly, age-friendly urban planning [20] might increase feelings of security [21] social connectedness and belonging [22]. Thirdly, driving cessation [23] increases the needs of older adults to be supported in their community mobility. Fourth, financial means [24] and health limitations [25] influence how older adults manage their community mobility needs. Interestingly, Musselwhite and Scott [24] report that older adults tend to focus on infrastructure barriers and enablers regarding their mobility, rather than aspects related to age stigma or social connectedness. However, if these needs are unmet, community mobility and social participation would be compromised.

Health equity means having a just and fair opportunity to achieve optimal health, thus addressing the health disparities that affect marginalised or excluded groups [26]. It is the authors’ view that unmet community mobility needs lead to health inequity. In addition to being unable to access healthcare facilities, older persons who are unable to access their life space may experience restrictions in meaningful activities, lose their sense of belonging to their communities and become socially isolated.

At the time of this review, the health equity characteristics associated with unmet community mobility needs for older persons were unknown. As the pandemic necessitated global adjustments to a new normal [5], governments, health authorities, transport providers, and commuters were called on to collaborate in taking efficient, sustainable and equitable transportation and mobility actions [27]. The authors from Karolinska Institutet, Stellenbosch University, Malmo University, University of Applied Sciences and Arts Western Switzerland and the University of the Witwatersrand formed a collaboration under the auspices of the South Africa – Sweden University Forum (SASUF). The aim of the scoping review was to determine the health equity characteristics of research where the reviewers found evidence of unmet needs of community mobility for older adults published between 2015 and April 2020 as informed by the PROGRESS-Plus framework [28].

## Methods

A preliminary rapid search for existing scoping, and systematic reviews, was conducted using Google Scholar with Stellenbosch University as library link. No similar reviews were noted. The scoping review followed the Johanna Briggs Institute (JBI) scoping review framework [29] and the PRISMA-Equity guidelines [30]. The stages of the scoping review are elaborated on in Table 1. Stage 1 included development of the scoping review protocol which is available from the corresponding author. A specialist librarian from Stellenbosch University assisted with Stage 2 (Search string development). Under the guidance of this librarian databases were selected and decision of such finalised during group discussion. The librarian was available for advice and support throughout Stage 3 (Database searches). Mendeley [31] was used in Stage 3 to collate full texts of articles. Search results were loaded into Covidence software [32] for managements and auditing of the study selection (Stage 4) and data extraction (Stage 5) processes. The data extraction sheet that was developed and used in Covidence is included as Supplementary file 1. The extracted data was summarized and interpreted during Stage 6. For qualitative data analysis, Taguette [33] an open-source tool for qualitative research, was used. For the quantitative data analysis, the Statistical Package for the Social Sciences version 27.0 (SPSS) was used [34].

**Table 1:** Stages, actions, and timeline of the scoping review

Scoping review stages	Actions taken	Timeline
Stage 1. Developing the scoping review	Develop scoping review questions, aim, inclusion and exclusion criteria, search strategy and draw up a protocol. Prisma-E guidelines are followed	4 – 25 February 2020
Stage 2. Defining and aligning the search strings, key, and index words	Iterative interaction with scoping aim, databases, and literature. Defining, test running, correcting, and finalising the search strings	26 February – 10 March 2020
Stage 3. Search the evidence	Searched were run on the following data bases: PubMed / MEDLINE, Scopus / Embase, CINAHL, PsycINFO via OVID and Web of Science	11 March – 17 April 2020
Stage 4. Study selection	Screening of 20 articles by the full research team led to confirmation of exclusion-inclusion criteria Thereafter, title and abstract screening, and then full text screening took place. During each screening stage, each article was reviewed by two authors. Conflicts were resolved through discussion between the research pair, or a third researcher if consensus was not reached	7 April – 23 November 2020
Stage 5. Data Extraction and Charting	Data was extracted, using a custom-made template that focuses on the aims of the review and PROGRESS-Plus equity framework characteristics [Cochrane Methods, 2019]. Charted data was extracted into Excel	16 October – 23 November 2020
Stage 6. Summarizing and interpreting the data	Quantitative data analysed using SPSS descriptive statistics Qualitative data is extracted, and data is imported into an open-source data analysis tool	18 January 2020 – 15 February 2021
Stage 7. Interpreting and dissemination of the results	The results were interpreted, written into a scoping review journal article, and submitted for publication to a peer review journal	11 March 2021 – 12 November 2021

The authors collaborated in all aspects of the scoping review in virtual bi-monthly meetings for group discussion, consolidation, and coordination of actions. A detailed decision-and-progress report was kept throughout the process.

### Eligibility criteria

Studies were included if they were primary research published in English between 2016 and 2020 comprising quantitative and qualitative research paradigms.The start date was selected to coincide with the promulgation of the UN’s Sustainable Development agenda. Participants had to be community dwelling, and 55 years and older. Studies did not have to have the specific aim of identifying or describing unmet community mobility needs. Studies were excluded if the unmet community mobility needs were identified in the home of participants exclusively.

### Search strategy

Medical Subject Headings (MeSH), a National Medical Library (NML) thesaurus that assists with the building and refining of search strings, key and index words with Boolean operators and the Participant, Context, Concept (PCC) were used to develop the following search strings:

(“older adults” OR “older people” OR “elderly people” OR “ageing people” OR “senior adults” OR “mature adults” OR “later life” OR retire* OR pension* OR elder* OR aged OR ageing OR seniors OR elders OR gerontol*).

AND

(community mobility OR “movement outside" OR “travel needs” OR "leisure activities" OR "social participation" OR “ageing in place” OR "Human Activities"[Mesh] OR transportation [mesh] OR transport* OR “transport poverty” OR travel OR recreation OR relaxation OR “Instrumental Activities of Daily Living” OR “Independent Living” OR “public transport” OR walk* OR drive* OR cycle* OR buss* OR train* OR “designated transport” OR “universal design”).

AND

(needs OR “unmet needs” OR challenges OR difficulties OR issues OR experiences OR wants OR “suppressed mobility”).

In addition, the following database specific restrictors were used:

- Pub Med/Medline: Publication date from 2016/01/01 to 2020/12/31. Humans. English. Abstracts Available. Core clinical journals. Age 55 + years.
- Scopus/ Embase: Source type – Journal articles. Date – 2016 to 2020. Subject Area – Social Science, Health professions, Psychology. Aged. Human. Language – English.
- CINAHL: Abstract available. Published date 20,160,101–20,201,231. Research article. Journal subset: Allied Health. Language: English. Age 55 + .
- PsycINFO via OVID: Middle age 40 to 64 years or aged 65 years and older or very old 85 years and older.
- Web of Science: Time span 2016 – 2020.

### Selection process

The first 20 included articles were screened together by all authors to refine selection criteria and thus improve inter-rater reliability. During this process reasons for excluding articles were inductively developed and imported into Covidence. Authors then commenced with blinded Title and Abstract Screening, with two consistent votes moving the screened article into Full Text Screening or exclusion. Conflicting votes were resolved in discussion between the authors who voted. Full Text Screening ensued using the same format and selection criteria.

### Data extraction and analysis

The Covidence Data Extraction Template was developed (see Supplementary file 1) and used by all authors within the Covidence program. The PROGRESS acronym is a useful framework for applying an equity lens in research [35], and includes place of residence, race/ethnicity/culture/language, occupation, gender and/or sex, religion, education, socio-economic status and social capital as some of the factors that are associated with health disparity. Additional health equity characteristics adopted from PROGRESS-Plus included personal characteristics that could be associated with discrimination (i.e. age, level of disability and HIV status), features of relationships and health habits (i.e. marital status and smoking status), and time-dependent factors that may cause disadvantage or risk to health (i.e. leaving hospital) [36].

The quality of the published research was not critically appraised but the research type, its statement of intent, main conclusion, sample size, sampling method, study design, data collection process and the type of analysis used were extracted to examine methodological tendencies and any possible impact on equity. All identified unmet community needs were extracted under the framework: physical accessibility, cost, availability, safety and other.

The authors divided into qualitative and quantitative analysis teams according to their research experience and strengths. At the conclusion of the data extraction all quantitative extracted data was moved from Excel to SPSS for analysis. Quantitative data were analysed predominantly with frequencies/percentages. First, we analysed frequency of reporting of each equity criteria and additional items on the data extraction form. Then we collapsed the criteria into content categories including study approach/ design (4 items), sample size (6 items), geographical area where research was conducted (6 items), and four domains known to influence community mobility of older adults: Gender, Disability, Education or socio-economic status, and Transport/Driving status (described in any form). For each category, we analysed the frequency. Inductive content analysis was undertaken to identify unmet community mobility needs from the findings, discussion and conclusions sections of included articles, using Taguette [33]. The authors individually read and inductively coded the data, creating provisional categories. During group discussions these categories were refined, and themes identified by consensus.

## Results

### Study selection

**Fig. 1:**
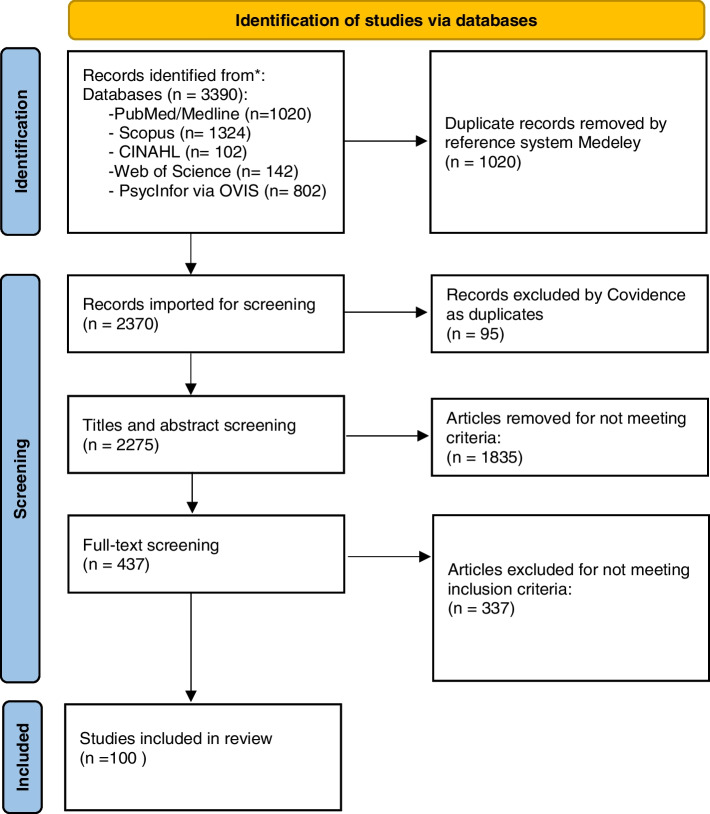
PRISMA 2020 flow diagram of the scoping review’s evidence selection

The results of the evidence selection phase are shown in a PRISMA 2020 flow diagram [37] as Fig. 1.

### Study characteristics

103 articles were identified for data extraction after full text screening. Three of the articles were removed by consensus when no unmet community mobility needs were identified during data extraction. The 100 remaining articles are listed as a supplementary file (Supplementary file 2). Most articles originated from the United States of America (USA) and Canada [39%], followed by Europe (27%), Asia including Turkey (19%), and Australia and New Zealand (11%). Only a few articles were reported from the middle and south Americas (3%) or Africa (1%). A summary of the articles in terms of research design, sample, and origin of study is presented in Table 2.

**Table 2:** Characteristics of the included articles for the review (*N* = 100)

Study Approach	n/100 (%)
Quantitative	43
Qualitative	33
Mixed methods	22
Not reported	2
Sample size	
Articles with sample size less than 10 participants	7
10–99 participants	39
100–499 participants	20
500–999 participants	6
1000 participants or more	24
Missing information on sample size	4
Geographical area	
North America	39
Middle and South America	3
Europe	27
Asia and Turkey	19
Australia and NZ	11
Africa	1

The settings in which study populations resided were not indicated in 38% of articles. Articles that did report this were predominantly done in urban settings (*N* = 28) described as urban (*n* = 20), city (*n* = 4), inner-city (*n* = 2), metropolitan (*n* = 1) and semi-urban (*n* = 1). Eight (8%) articles considered populations from both urban and rural contexts and six (6%) articles were done in rural settings. The remaining articles reported their study areas to be regions, districts, provinces, greater municipalities, or retirement homes.

In group discussions the authors identified four equity variables that were known to impact on community mobility of older adults; Gender, Disability, Education or socio-economic status, and Transport/Driving (described in any form). Only 10 (10%) of the included articles described all four of these equity variables. Overall, the targeted health equity characteristics were underreported in most of the analysed articles, as shown in Table 3, although most of the papers reported some of them.

**Table 3:** Articles that reported equity characteristics (*N* = 100)

	Reported in any form		Total
Health equity characteristics	Yes	No	
1. Gender/Sex	65	35	100
2. Education or socioeconomic status	51	49	100
3. Diagnosis/disability/health status	40	60	100
4. Driving status	38	62	100
5. Sexual orientation	2	98	100
6. Marital status	27	73	100
7. Living arrangements	35	65	100
8. Race/ethnicity/culture/religion	38	62	100
All characteristics 1–4 above are reported	10	90	100

### Factors restricting older adults’ ability to meet their community mobility needs

Older adults’ ability to meet their community mobility needs is affected by a complex set of factors that are both external and internal. These represent the multifactorial interaction between the person and their environment, when engaging in community mobility as an occupation. Six themes were developed to capture these factors. External factors included societal attitudes, the built environment, and service availability. In contrast, internal factors included finances, personal fear and apprehension, and personal functional limitations.

#### Built environment

Environmental barriers that limited older adults’ community mobility were wide-ranging. Aesthetics were mentioned as having a positive [38, 39] and a negative [40] impact on community mobility and mostly referred to parks, vegetation, and greenery [41, 42]. More often, the experience was negative and related to neglect or lack of maintenance [43]. The latter was experienced not only as unpleasant but also decreased the sense of security [44]. Dirt, litter and graffiti was also mentioned as aesthetics that had a negative impact on feeling safe in relation to community mobility needs [43]. The importance of the environment being a positive experience [45], having flat surfaces, and comfortable places to rest [46] was reported as a need.

Some features of environmental design such as flat, non-slippery surfaces directly improved community mobility [42]. Conversely, community mobility limitations were linked with faulty design features and often associated with rural environments [47–49]. Rural and urban environments were compared in several articles [42, 47–53], with rural environments having more factors deemed undesirable in terms of accessibility, safety, availability of services and cost considerations. Some neighbourhoods reported city planning barriers and older adults living there experienced as being cut-off or cordoned from other parts of the city [43], bringing a feeling of living in a ghetto and making traveling to other parts of the city difficult.

#### Service availability

The availability of public transport services often shaped the community mobility of older adults [54–57] Available services were not always accessible and not frequent enough [46, 58, 59], pick up and drop off points were too far away to walk to, or the services followed an unpredictable schedule [38]. Additional issues reported were busses passing without stopping at rush hour [43], overcrowding and unavailable seat reservation [54], and unannounced route changes [60]. The time spent travelling on public transport was described as a waste of time [45].

The availability of services was also affected by support services and facilities such as the clearing of snow and protection against harsh weather [48], the lack of parking space [46], poor road maintenance [61] and poorly staffed services [61]. Living in rural areas was noted as being more severely affected by service availability and supportive services [58]. Suggested solutions were offered such as offering free travel passes to older adults [62], Requesting family or friends to assist with transportation was indicated as a strategy used [47, 63] but this was found to pose interpersonal and social problems as families were not always available and the fear of imposing affected the solution [47, 64].

#### Societal attitudes

Unmet community mobility needs due to societal attitudinal factors were experienced in persons growing older with a LGBTQ orientation [52]. Being female [63] and having a disability [50] were also reported as a barrier in community mobility. Older women reported having less opportunities to travel than their male counterparts [55, 63, 65]. Finally, not being able to apply adaptive strategies for a driving cessation led to a loss of independence [66].

Family attitudes also imposed restrictions on older adults to go to out of home places due to various concerns [67]. Non-driving older adults depended on family members for transport [63, 64] but the availability and willingness of families to assist affected the possibility [47]. Personal attitudes also affected older adults’ community mobility and they reported disliking being dependent on others for transport [67].

In addition, older adults reported being concerned and inhibited to use public transport due to attitudes of public transport operators and fellow commuters [55, 68]. This was reported to be problematic when they were boarding, disembarking or finding a seat [43, 67, 69].

#### Personal financial constraints

Low income was indicated as a substantial barrier to transportation and community access for older adults [70] and applied especially so to rural older adults [49]. Whereas vehicle ownership had a positive impact that significantly increased the trip making of older adults [55] it was also the most expensive form of mobility for them [71]. Personal financial constraints limited the upkeep [43] and running cost of vehicles, which affected ownership of motor vehicles [72], motorcycles [55] and bicycles [73]. This necessitated older adults to consider pay-per-use transportation options such as rickshaws, taxis and auto share opportunities [67]. Conversely, the number of modal options available [58] and levels of satisfaction with the quality of public transport [62] reduced relative to income levels.

Financial restrictions also led to increased self-regulation [67] and resulted in older adults taking fewer and shorter trips [74] thus resulting in fewer out of home activities [75]; This increased their risk for social exclusion [76] and negatively affected their health seeking behaviour [61]. Interventions to address transportation-disadvantages of older adults were shown to be problematic. In some countries reimbursement for travel was offered but this was less than the actual cost of travelling for older adults [50] and computing the cost per passenger kilometre of the shared fleet concept was shown to be comparable to private car ownership [71]. Evidence showed that access to affordable, adequate transportation is compromised through social and political forces, which marginalise historically disadvantaged populations [70].

#### Personal fear and apprehension

Older adults expressed multiple fears related to community mobility and such apprehension extended to driving, walking, using public transport and cycling mobility [55] [76–78]. Enabling community mobility factors reported were being familiar with the area and having company with whom to undertake out of home mobility [79]. Two dominant fears were fear of crime and the fear of falling.

The fear of crime was evident in several articles [42, 51, 55, 73, 76, 80, 81]. Loukaitou-Sideris [43], Lee [82] and Klicnik [73] reported older adults felt unsafe because of other people they could see in the environment who were up to mischief, drunk, homeless, or dealing or taking drugs. The fear of being robbed was noted [55], as was a fear of being taken advantage of [43]. As a result, older adults reported avoiding walking at night [43, 78]. Busy streets were highlighted as a threat to feelings of being in control [78, 80] and not feeling safe [51]. The fear of crime also seemed to be related to the fear of falls; for example, one participant in Loukaitou-Sideris [43] study reported difficulties in observing her surroundings for threats of crime, and the floor for trip and fall hazards, at the same time.

Fears of falling was regularly evident [42–44, 54, 67, 68, 80, 83]. This fear was related to walking [80] and using the bus [83]. Pedestrian infrastructure and traffic hazards were two key themes in the fear of falling while walking [43]. Older adults expressed concern about the condition of walking surfaces such as uneven pavements [43], loose tiles [80], broken steps [44], holes in the road or pavement surface [43, 44], high curb cuts [44], and surfaces becoming slippery when wet [80]. A lack of adequate street lighting was also a concern [44]. In addition, the available space for walking influenced the fear of falls. Space to walk was limited by litter [43, 44], garbage cans [44], homeless people and their pavement encampments [43], street vendor’s merchandise [43], parked cars [44, 80], and crowded roads [54]. Fear of falling due to traffic hazards included the extent to which other road users obeyed the traffic rules and the crossing of roads [42, 54]. The behaviour of other road users was also a concern for cyclists [77]. When using the bus, the fear of falls was primarily associated with the bus pulling off before older adults had an opportunity to sit down [68, 83].

#### Personal functional limitations

Personal functional limitations were identified as a factor that negatively impacted community mobility for older persons. Firstly, health issues and disability reduced transport options [84]. Mobility limitations prevented some older persons from accessing and using community resources such as parks [85] and public transport [46]. In addition, several studies [46, 86, 87] found that community mobility is increased for people who perceive their community as accessible and walkable, and they will be more willing to walk to access transport than those who live in communities perceived not to be accessible. Similarly, older persons who perceived their community resources within a 20-min walk from home to be accessible, walked more for recreation than those who lived in neighbourhoods perceived to have poor access to destinations [87]. Difficulty communicating with drivers [50] due to language barriers or impairments was also identified as a factor limiting their independent use of community resources and community mobility resources.

## Discussion

This scoping review aimed to determine the health equity characteristics of research describing the unmet needs of community mobility for older adults as informed by PROGRESS-Plus characteristics. It therefore performed two functions, firstly, to synthesise articles that directly or indirectly reported unmet community mobility needs of older adults and secondly, to provide a critical stance of the extent to which equity considerations were being reported.

The reporting of health equity characteristics was inconsistent in research exploring the unmet community mobility needs of older adults. Variability in the detail reported in articles contained in this review made it difficult to explore the equity characteristics of research undertaken to explore unmet community needs of older adults. We concede that these variables might have been considered during the research process, however, these were not consistently or uniformly reported. This results in difficulties to generalise the finding from various studies targeting unmet mobility needs, as important information regarding the health equity characteristics of the target samples were overall underreported. In addition, contextual factors which demonstrated direct impact on community mobility were also underreported, for example affordability of services was reported in only 25 of the 100 papers.

Furthermore, the characteristics of participants that might experience marginalisation, which could impact their community mobility, was also under reported. While binary gender categories [male, female] were moderately well reported [65%] none reported on non-binary gender categories; thus, silencing factors that potentially impact on the experiences of LGBTQIA + community. Similarly, sexual orientation was reported in only two articles. Many reporting guidelines are now available for the consistent writing up of different types of articles. Better use of existing reporting guidelines is therefore strongly recommended based on this scoping review. The use of Progress Plus [28] criteria worked particularly well for our review.

Countries from which articles originated were predominantly higher income countries, with North America dominating, followed by Europe producing the most articles [see Table 2]. The unmet community mobility needs of older adults living in Low- and Middle-Income Countries [LMCI] were largely underrepresented with only one source from Africa and three from the middle and south Americas (3%). Bearing in mind prevailing lower socio-economic conditions in these countries expected to impact on community mobility infrastructure, resources and services causing transport poverty—defined as the interrelation result of a systemic lack of transport services and related infrastructure, accessibility difficulties, affordability of available transport and disproportionate exposure to negative transport externalities [88] we expect a higher incidence of unmet community mobility needs.

Considering the urban – rural debate, articles predominantly focussed on urban settings [*n* = 28] or both urban and rural [*n* = 3] with only six exploring rural environments [*n* = 6]. Under-reporting of lower resource contexts are particularly problematic because the small number of articles that included data collection in rural areas highlighted a range of factors causing transport poverty [89]. An article from Uganda, Africa reported the poor condition of roads, long traveling times, poor public infrastructure, unavailable or costly transport impacting impact the community mobility of older adults [61].

There were external and internal factors reported to affect community mobility. External factors, outside the control of older adults were societal attitudes, built environments, and the availability of services. Internal factors such as, personal financial constraints, fear and apprehension, and functional limitations were factors over which older adults may have more influence. These interrelated factors point to a need for integrated policies and multi-agency services that support the community mobility of older adults. This is particularly important in low and middle income countries where population aging is occurring faster than in high income countries, in a context of lower levels of industrialisation and wealth development [90].

A range of attitudes with direct negative impact on the community mobility of older adults emerged and are detailed in the results. Attitudinal barriers pertaining to families, transport operators and fellow commuters impacted the more vulnerable groups, especially persons with disability, women and members of the LGBTQIA + community. More research is required to explore the impact of societal attitudes on the community mobility of older adults. Research that acknowledges the unique needs of these groups but also recognises the differences within such communities as they age [91].

Concerns around personal safety and fear, which included the fear of falling, social embarrassment and getting lost, emerged as a strong factor impacting community mobility of older adults with high prevalence across different socio-economic contexts and geographical regions. Older adults also seem to be particularly susceptible to their environment, which had a direct impact on their community mobility. Environmental considerations emerged strongly as an influence on and was highlighted by older adults as a priority. Consequences of the built environment on community mobility needs limited access to health care, goods and services, isolating older adults from familiar lifestyle habits and social networks. The importance of an aesthetic environment and its link to community mobility is of interest, yet often overlooked. This could be linked to persons living in high density living arrangements [41].

The review followed hegemonic practices with articles focussing almost exclusively on the unmet community mobility needs of older adults reported in peer reviewed articles detailing the needs of older adults that are considered relatively easy to reach. This places us at risk of making recommendations or developing interventions that only meet the needs of a small part of the older adult’s population.

### Limitation of the study

The term "unmet needs" is difficult to define clearly and might have been interpreted differently by members of the research team. There are a wide range of unmet community mobility needs that are interrelated. Despite the use of dual reviewers and consensus strategies were used for both stages of the review process, articles might have been missed in the process.

Articles might have been missed because only English-language articles were included in the review. Research reported in other formats and/or languages might have yielded additional findings. This might also have contributed to the findings that some areas, specifically those from non-English areas, were not represented in this scoping review.

The additional use of hand-searching strategies, inclusion of unpublished and/or publications that had not been peer-reviewed would have further broadened the search and might have impacted on the findings obtained. For example, it is not known whether more sources might have been obtained from Low- and Middle- Income Countries (LMCI) had such a strategy been used. We therefore recommend that further reviews include grey literature in LMCI countries.

## Conclusion

The reporting of unmet community mobility needs was found to be inconsistent and excluding several groups of older adults considered vulnerable as such the knowledgebase was found to be limited. The lack of systematic information regarding health equity characteristics of the samples severely limited the generalizability, but also the conclusions that can be drawn from these studies. The scoping review highlights the need for a consistent and more detailed reporting standard of studies on older adults to increase health equity. Gender, disability, education, and community mobility / driving were identified as key health equity characteristics, that should be reported in all community mobility studies of older adults.

Older adults in the reported articles showed unmet community mobility needs in relation to physical functioning, social attitudes, physical accessibility of built environments, lack of availability of services, high costs with lack of personal finances and fear of crime and falls. The complexity and multi-sectoral nature of these needs, require interprofessional approaches and research to explore the full extent of barriers and possible solutions. This is further complicated by the current nature of evidence creation and dissemination as well as the divide between sectors.

The equity focus of this scoping review revealed a skewed representation of primary researched evidence, favouring mainstream population groups, the global north, and urban contexts. Findings cannot be assumed to be representative of all older adults. The likelihood of unmet needs that have not been identified for particular populations is high. As such, the available evidence on unmet community needs of older adults should not be considered complete. Future research should consider a global drive to ensure a comprehensive and equitable approach to addressing factors that affect the community mobility needs of older adults. Researchers need to find a balanced approach in reporting health equity characteristics reflecting the diversity of the participants. We suggest that the characteristics that best illuminate the research question are prioritised. Research that focuses on the creation of evidence that will leave no one behind.

## Supplementary Information

**Additional file 1. **Data extraction template.

**Additional file 2: Supplementary file 2.** List of articles included in the scoping review.

## Data Availability

The data supporting the conclusion of this article is included within the article and its additional files.
