# Correction: The health equity characteristics of research exploring the unmet community mobility needs of older adults: a scoping review

**DOI:** 10.1186/s12877-022-03600-8

**Published:** 2023-01-12

**Authors:** Hester van Biljon, Lana van Niekerk, Isabel Margot-Cattin, Fasloen Adams, Nicola Plastow, David Bellagamba, Anders Kottorp, Ann-Helen Patomella

**Affiliations:** 1 Stellenbosch University, Stellenbosch, South Africa; 2 University of Applied Sciences and Arts, Windisch, Switzerland; 3 Malmö University, Malmö, Sweden; 4 Karolinska Institutet, Solna, Sweden

**Correction: BMC Geriatr 22, 808 (2022)**

**https://doi.org/10.1186/s12877-022-03492-8**

After publication of this article [1], the authors reported that in the abstract, paragraph Conclusions, the 3rd sentence should not start with “15”, but with “Fifteen years”.

The original article [1] has been corrected.
