# Supplementary material for: The health equity characteristics of research exploring the unmet community mobility needs of older adults: a scoping review
Source: BMC Geriatr. 2022 Oct 20;22:808. doi: 10.1186/s12877-022-03492-8 (PMC9585759; doi:10.1186/s12877-022-03492-8)
Supplement: Supplementary file 1 — Additional file 1. Data extraction template. [file 12877_2022_3492_MOESM1_ESM.docx]

Supplementary file 1: Data extraction template

The Covidence Data Extraction Template was adapted to meet the scoping reviews specific aim and the table below shows the final data extraction instrument that was used.

COVIDENCE Data Extraction Template: Community Mobility Needs of Older Adults

| **Article** | **Extraction** | **Comparison** |
| --- | --- | --- |
| **Authors** |  |  |
| **Year of publication** |  |  |
| **Covidence No** |  |  |
| **Geographic region** |  |  |
| **Statement of intent of the study [aim/purpose/objective/research question]** |  |  |
| **Main conclusion drawn from the study** |  |  |
| **Identified unmet community mobility needs [physical accessibility]** |  |  |
| **Identified unmet community mobility needs [cost]** |  |  |
| **Identified unmet community mobility needs [availability]** |  |  |
| **Identified unmet community mobility needs [safety]** |  |  |
| **Identified unmet community mobility needs [other]** |  |  |
| **Sample size [n]** |  |  |
| **Female** |  |  |
| **Male** |  |  |
| **Other gender** |  |  |
| **Age** |  |  |
| **Age range [lowest]** |  |  |
| **Age range [highest]** |  |  |
| **Age groups** |  |  |
| **Sampling method** |  |  |
| **Place of residence [a]** |  |  |
| **Place of residence [b]** |  |  |
| **Race / ethnicity / culture / language / religion** |  |  |
| **Work status** |  |  |
| **Education** |  |  |
| **Socio-economic status** |  |  |
| **Social capital** |  |  |
| **Diagnosis OR health conditions OR impairments** |  |  |
| **Disability** |  |  |
| **Sexual orientation** |  |  |
| **Marital status** |  |  |
| **Living arrangement** |  |  |
| **Driving status** |  |  |
| **Study design** |  |  |
| **Data collection process** |  |  |
| **Type of study conducted** |  |  |
| **Type of analysis used** |  |  |
