# Supplementary material for: The health equity characteristics of research exploring the unmet community mobility needs of older adults: a scoping review
Source: BMC Geriatr. 2022 Oct 20;22:808. doi: 10.1186/s12877-022-03492-8 (PMC9585759; doi:10.1186/s12877-022-03492-8)
Supplement: Supplementary file 2 — Additional file 2: Supplementary file 2. List of articles included in the scoping review. [file 12877_2022_3492_MOESM2_ESM.docx]

**Supplementary file 2: List of articles included in the scoping review titled:**

***The health equity characteristics of research exploring the unmet community mobility needs of older adults: A scoping review***

| **N=100** | **Article included in the review references in alphabetical order of the first author surname** |
| --- | --- |
| 1 | Aceves-González, C., May, A., & Cook, S. (2016). An observational comparison of the older and younger bus passenger experience in a developing world city. Ergonomics, 59(6), 840–850. <https://doi.org/10.1080/00140139.2015.1091513> |
| 2 | Adorno, G., Fields, N., Cronley, C., Parekh, R., & Magruder, K. (2018). Ageing in a low-density urban city: Transportation mobility as a social equity issue. Ageing and Society, 38(2), 296–320. <https://doi.org/10.1017/S0144686X16000994> |
| 3 | Ahmad, Z., Batool, Z., & Starkey, P. (2019). Understanding mobility characteristics and needs of older persons in urban Pakistan with respect to use of public transport and self-driving. Journal of Transport Geography, 74, 181–190.  <https://doi.org/10.1016/j.jtrangeo.2018.11.015> |
| 4 | Alidoust, S., Bosman, C., & Holden, G. (2018). Talking while walking: an investigation of perceived neighbourhood walkability and its implications for the social life of older people. Journal of Housing and the Built Environment, 33(1), 133–150.  <https://doi.org/10.1007/s10901-017-9558-1> |
| 5 | Alidoust, S., Bosman, C., & Holden, G. (2019). Planning for healthy ageing: How the use of third places contributes to the social health of older populations. Ageing and Society, 39(7), 1459–1484. <https://doi.org/10.1017/S0144686X18000065> |
| 6 | Ariza-Álvarez, A., Arranz-López, A., & Soria-Lara, J. A. (2019). Comparing walking accessibility variations between groceries and other retail activities for seniors. Research in Transportation Economics.  <https://doi.org/10.1016/j.retrec.2019.100745> |
| 7 | Bengtson, V., Endacott, C., & Kang, S. (2018). Older adults in churches: Differences in perceptions of clergy and older members. Journal of Religion, Spirituality & Aging, 30(2 PG-154–178), 154–178. <http://dx.doi.org/10.1080/15528030.2017.1414727> |
| 8 | Beyazova, M., Doğan, A., Kutsal, Y. G., Karahan, S., Arslan, Ş., Gökkaya, K. O., Toraman, F., Dinçer, N., Hizmetli, S., Şenel, K., Yazgan, P., Ortancıl, Ö., İrdesel, J., Özyemişçi-Taşkıran, Ö., Borman, P., Okumuş, M., Ceceli, E., Evcik, D., Ay, S., & Öztop, P. (2020). Environmental characteristics of older people attending physical medicine and rehabilitation outpatient clinics. Central European Journal of Public Health, 28(1), 33–39.  <https://doi.org/10.21101/cejph.a5194> |
| 9 | Boakye-Dankwa, E., Barnett, A., Pachana, N. A., Turrell, G., & Cerin, E. (2019). Associations between latent classes of perceived neighborhood destination accessibility and walking behaviors in older adults of a low-density and a high-density city. Journal of Aging and Physical Activity, 27(4), 553–564. <https://doi.org/10.1123/japa.2018-0297> |
| 10 | Bozdağ, A., Gümüş, M. G., Gümüş, K., & Durduran, S. (2017). Accessibility analysis for the elderly in an urban area from Turkey. Transylvanian Review of Administrative Sciences, 2017, 21–37. <https://doi.org/10.24193/TRAS.SI2017.2> |
| 11 | Bradford, J. B., Putney, J. M., Shepard, B. L., Sass, S. E., Rudicel, S., Ladd, H., & Cahill, S. (2016). Healthy Aging in Community for Older Lesbians. LGBT Health, 3(2), 109–115. <https://doi.org/10.1089/lgbt.2015.0019> |
| 12 | Brown, D. L., Glasgow, N., Kulcsar, L. J., Sanders, S., & Thiede, B. C. (2019). The multi-scalar organization of aging-related services in US rural places. Journal of Rural Studies, 68, 219–229. <https://doi.org/10.1016/j.jrurstud.2018.09.010> |
| 13 | Choi, M., O’Connor, M. L., Mingo, C. A., & Mezuk, B. (2016). Gender and racial disparities in life-space constriction among older adults. The Gerontologist, 56(6 PG-1153–1160), 1153–1160. <https://doi.org/http://dx.doi.org/10.1093/geront/gnv061> |
| 14 | Choi, N. G., & DiNitto, D. M. (2016). Depressive symptoms among older adults who do not drive: Association with mobility resources and perceived transportation barriers. The Gerontologist, 56(3 PG-432–443), 432–443.  <https://doi.org/http://dx.doi.org/10.1093/geront/gnu116> |
| 15 | Curl, A., Thompson, C. W., Aspinall, P., & Ormerod, M. (2016). Developing an audit checklist to assess outdoor falls risk. Proceedings of the Institution of Civil Engineers: Urban Design and Planning, 169(3), 138–153.  <https://doi.org/10.1680/udap.14.00056> |
| 16 | Dare, J., Wilkinson, C., Marquis, R., & Donovan, R. J. (2018). “The people make it fun, the activities we do just make sure we turn up on time.” Factors influencing older adults’ participation in community-based group programmes in Perth, Western Australia. Health and Social Care in the Community, 26(6), 871–881.  <https://doi.org/10.1111/hsc.12600> |
| 17 | De Waard, D., Lambers, A. A. A., & Brookhuis, K. A. (2016). Crossing time of older cyclists at signalised junctions. International Journal of Human Factors and Ergonomics, 4(1), 1–9. <https://doi.org/10.1504/IJHFE.2016.076561> |
| 18 | Duim, E., Lebrão, M. L., & Antunes, J. L. F. (2017). Walking speed of older people and pedestrian crossing time. Journal of Transport and Health, 5, 70–76.  <https://doi.org/10.1016/j.jth.2017.02.001> |
| 19 | Emirhafizović, M., & Šadić, S. (2018). Ageing in the Urban Context: Evidence from Sarajevo. Socialni Studia/Social Studies, 15(1), 85–98.  <https://doi.org/10.5817/soc2018-1-85> |
| 20 | Ferris, R. E., Glicksman, A., & Kleban, M. H. (2016). Environmental predictors of unmet home- and community-based service needs of older adults. Journal of Applied Gerontology, 35(2 PG-179–208), 179–208.  <https://doi.org/http://dx.doi.org/10.1177/0733464814525504> |
| 21 | Fitt, H., Curl, A., Dionisio, M. R., Ahuriri-Driscoll, A., & Pawson, E. (2019). Considering the wellbeing implications for an ageing population of a transition to automated vehicles. Research in Transportation Business and Management, 30.  <https://doi.org/10.1016/j.rtbm.2019.100382> |
| 22 | Franke, T., Winters, M., McKay, H., Chaudhury, H., & Sims-Gould, J. (2017). A grounded visualization approach to explore sociospatial and temporal complexities of older adults’ mobility. Social Science and Medicine, 193, 59–69.  <https://doi.org/10.1016/j.socscimed.2017.09.047> |
| 23 | Gao, J., & Kerstetter, D. L. (2016). Using an intersectionality perspective to uncover older Chinese female’s perceived travel constraints and negotiation strategies. Tourism Management, 57, 128–138. <https://doi.org/10.1016/j.tourman.2016.06.001> |
| 24 | Ghani, F., Rachele, J. N., Loh, V. H., Washington, S., & Turrell, G. (2018). Do differences in built environments explain age differences in transport walking across neighbourhoods? Journal of Transport and Health, 9, 83–95.  <https://doi.org/10.1016/j.jth.2018.03.010> |
| 25 | Gould, C. E., Shah, S., Brunskill, S. R., Brown, K., Oliva, N. L., Hosseini, C., Bauer, E., & Huh, J. W. T. (2017). RESOLV: Development of a telephone-based program designed to increase socialization in older veterans. Educational Gerontology, 43(8), 379–392.  <https://doi.org/10.1080/03601277.2017.1299522> |
| 26 | Hagan, R. J. (2019). Getting out of the house: The use of community transport as a third place for rural-dwelling older adults. Ageing and Society.  <https://doi.org/10.1017/S0144686X19000722> |
| 27 | Harvey, J., Guo, W., & Edwards, S. (2019). Increasing mobility for older travellers through engagement with technology. Transportation Research Part F: Traffic Psychology and Behaviour, 60(Wakefield, J. (2015). The generation that tech forgot. URL: Accessed 14 Nov 2017. PG-172-184), 172–184. <https://doi.org/http://dx.doi.org/10.1016/j.trf.2018.10.019> |
| 28 | He, S. Y., Cheung, Y. H. Y., & Tao, S. (2018). Travel mobility and social participation among older people in a transit metropolis: A socio-spatial-temporal perspective. Transportation Research Part A: Policy and Practice, 118, 608–626.  <https://doi.org/10.1016/j.tra.2018.09.006> |
| 29 | Heatwole Shank, K. S., Kenley, B., Brown, S., Shipley, J., Baum, M., & Beers, C. (2020). “We need more things for us”: Being low income and underoccupied in older age. Canadian Journal of Occupational Therapy, 87(1), 21–29.  <https://doi.org/10.1177/0008417419838360> |
| 30 | Henning-Smith, C. E., Gonzales, G., & Shippee, T. P. (2016). Barriers to Timely Medical Care for Older Adults by Disability Status and Household Composition. Journal of Disability Policy Studies, 27(2), 116–127.  <https://doi.org/10.1177/1044207316637547> |
| 31 | Hong, A., Sallis, J. F., King, A. C., Conway, T. L., Saelens, B., Cain, K. L., Fox, E. H., & Frank, L. D. (2018). Linking green space to neighborhood social capital in older adults: The role of perceived safety. Social Science and Medicine, 207, 38–45.  <https://doi.org/10.1016/j.socscimed.2018.04.051> |
| 32 | Hulko, W., Mirza, N., & Seeley, L. (2020). Older Adults’ Views on the Repositioning of Primary and Community Care. Canadian Journal on Aging.  <https://doi.org/10.1017/S0714980819000540> |
| 33 | Hwang, J., Wang, L., Siever, J., Del Medico, T., & Jones, C. A. (2019). Loneliness and social isolation among older adults in a community exercise program: A qualitative study. Aging & Mental Health, 23(6 PG-736–742), 736–742.  <https://doi.org/http://dx.doi.org/10.1080/13607863.2018.1450835> |
| 34 | Ioannou, B. (2019). Ageing in suburban neighbourhoods: Planning, densities and place assessment. Urban Planning, 4(2 TheCity Aging and Urban Planning), 18–30.  <https://doi.org/10.17645/up.v4i2.1863> |
| 35 | Karekla, X., & Tyler, N. (2019). Reducing non-collision injuries aboard buses: Passenger balance whilst climbing the stairs. Safety Science, 112, 152–161.  <https://doi.org/10.1016/j.ssci.2018.10.023> |
| 36 | Keeney, T., & Jette, A. M. (2019). Individual and Environmental Determinants of Late-Life Community Disability for Persons Aging with Cardiovascular Disease. American Journal of Physical Medicine & Rehabilitation, 98(1), 30–34. <https://doi.org/10.1097/PHM.0000000000001011> |
| 37 | Kim, D. (2019). The transportation safety of elderly pedestrians: Modeling contributing factors to elderly pedestrian collisions. Accident Analysis and Prevention, 131(Zegeer, C.K., Stutts, J.C., Huang, H., Zhou, M., Rodgman, E. (1993). Analysis of elderly pedestrian accidents and recommended countermeasures. Transp. Res. Rec. 1405, 56-63. PG-268-274), 268–274. <https://doi.org/http://dx.doi.org/10.1016/j.aap.2019.07.009> |
| 38 | King, M. J., & Scott-Parker, B. J. (2017). Older male and female drivers in car-dependent settings: How much do they use other modes, and do they compensate for reduced driving to maintain mobility? Ageing and Society, 37(6), 1249–1267.  <https://doi.org/10.1017/S0144686X15001555> |
| 39 | Klicnik, I., & Dogra, S. (2019). Perspectives on active transportation in a mid-sized age-friendly city: “you stay home.” International Journal of Environmental Research and Public Health, 16(24 PG-). <https://doi.org/10.3390/ijerph16244916> |
| 40 | Knight, A., Black, R., Whitsed, R., & Harvey, R. (2018). Enhancing the usability and benefits of open space for older people in regional Australia. Australian Planner, 55(2), 73–83. <https://doi.org/10.1080/07293682.2018.1521454> |
| 41 | Lai, M. M., Lein, S. Y., Lau, S. H., & Lai, M. L. (2016). Modeling Age-Friendly Environment, Active Aging, and Social Connectedness in an Emerging Asian Economy. Journal of Aging Research, 2016. <https://doi.org/10.1155/2016/2052380> |
| 42 | Lee, E., & Dean, J. (2018). Perceptions of walkability and determinants of walking behaviour among urban seniors in Toronto, Canada. Journal of Transport and Health, 9, 309–320. <https://doi.org/10.1016/j.jth.2018.03.004> |
| 43 | Lee, E. A. L., Same, A., McNamara, B., & Rosenwax, L. (2018). An Accessible and Affordable Transport Intervention for Older People Living in the Community. Home Health Care Management and Practice, 30(2), 54–60.  <https://doi.org/10.1177/1084822317744759> |
| 44 | Lehning, A., Kim, K., Smith, R., & Choi, M. (2018). Does economic vulnerability moderate the association between transportation mode and social activity restrictions in later life? Ageing and Society, 38(10), 2041–2060.  <https://doi.org/10.1017/S0144686X17000411> |
| 45 | Levasseur, M., Coallier, J. C., Gabaude, C., Beaudry, M., Bédard, M., Langlais, M. È., & St-Pierre, C. (2016). Facilitators, barriers and needs in the use of adaptive driving strategies to enhance older drivers’ mobility: Importance of openness, perceptions, knowledge and support. Transportation Research Part F: Traffic Psychology and Behaviour, 43, 56–66.  <https://doi.org/10.1016/j.trf.2016.09.014> |
| 46 | Loukaitou-Sideris, A., Wachs, M., & Pinski, M. (2019). Toward a Richer Picture of the Mobility Needs of Older Americans. Journal of the American Planning Association, 85(4), 482–500. <https://doi.org/10.1080/01944363.2019.1630295> |
| 47 | Luiu, C., Tight, M., & Burrow, M. (2018). An investigation into the factors influencing travel needs during later life. Journal of Transport and Health, 11, 86–99.  <https://doi.org/10.1016/j.jth.2018.10.005> |
| 48 | Marquet, O., Hipp, J. A., & Miralles-Guasch, C. (2017). Neighborhood walkability and active ageing: A difference in differences assessment of active transportation over ten years. Journal of Transport and Health, 7, 190–201.  <https://doi.org/10.1016/j.jth.2017.09.006> |
| 49 | Menec, V. H., Brown, C. L., Newall, N. E. G., & Nowicki, S. (2016). How Important is Having Amenities Within Walking Distance to Middle-Aged and Older Adults, and Does the Perceived Importance Relate to Walking? Journal of Aging and Health, 28(3), 546–567.  <https://doi.org/10.1177/0898264315597352> |
| 50 | Meurer, J., Stein, M., Randall, D., & Wulf, V. (2018). Designing for way-finding as practices – A study of elderly people’s mobility. International Journal of Human Computer Studies, 115, 40–51. <https://doi.org/10.1016/j.ijhcs.2018.01.008> |
| 51 | Mohd, S., Abdul Latiff, A. R., & Senadjki, A. (2019). Travel behavior of elderly in george town and Malacca, Malaysia. Sustainability (Switzerland), 11(19).  <https://doi.org/10.3390/su11195251> |
| 52 | Moran, M. R., Werner, P., Doron, I., HaGani, N., Benvenisti, Y., King, A. C., Winter, S. J., Sheats, J. L., Garber, R., Motro, H., & Ergon, S. (2017). Exploring the objective and perceived environmental attributes of older adults’ neighborhood walking routes: A mixed methods analysis. Journal of Aging and Physical Activity, 25(3), 420–431.  <https://doi.org/10.1123/japa.2016-0165> |
| 53 | Mullen, N. W., Parker, B., Wiersma, E., Stinchcombe, A., & Bédard, M. (2017). Looking Forward and Looking Back: Older Adults’ Views of the Impacts of Stopping Driving. Occupational Therapy in Health Care, 31(3), 188–204.  <https://doi.org/10.1080/07380577.2017.1337282> |
| 54 | Murray, A., & Musselwhite, C. (2019). Older peoples’ experiences of informal support after giving up driving. Research in Transportation Business and Management, 30(June), 100367. <https://doi.org/10.1016/j.rtbm.2019.100367> |
| 55 | Musselwhite, C., & Haddad, H. (2017). The travel needs of older people and what happens when people give-up driving. In Transport and Sustainability (Vol. 10, pp. 93–115).  <https://doi.org/10.1108/S2044-994120170000010003> |
| 56 | Nakanishi, H., & Black, J. A. (2016). Travel habit creation of the elderly and the transition to sustainable transport: Exploratory research based on a retrospective survey. International Journal of Sustainable Transportation, 10(7), 604–616.  <https://doi.org/10.1080/15568318.2015.1059526> |
| 57 | Navarrete-Reyes, A. P., Medina-Rimoldi, C. T., & Avila-Funes, J. A. (2017). Correlates of subjective transportation deficiency among older adults attending outpatient clinics in a tertiary care hospital in Mexico City. Geriatrics and Gerontology International, 17(11), 1893–1898. <https://doi.org/10.1111/ggi.12987> |
| 58 | Neville, S., Adams, J., Napier, S., Shannon, K., & Jackson, D. (2018). “Engaging in my rural community”: perceptions of people aged 85 years and over. International Journal of Qualitative Studies on Health and Well-Being, 13(1).  <https://doi.org/10.1080/17482631.2018.1503908> |
| 59 | Nordbakke, S. T. D. (2019). Mobility, out-of-home activity participation and needs fulfilment in later life. International Journal of Environmental Research and Public Health, 16(24). <https://doi.org/10.3390/ijerph16245109> |
| 60 | Ottoni, C. A., Sims-Gould, J., Winters, M., Heijnen, M., & McKay, H. A. (2016). “Benches become like porches”: Built and social environment influences on older adults’ experiences of mobility and well-being. Social Science and Medicine, 169, 33–41.  <https://doi.org/10.1016/j.socscimed.2016.08.044> |
| 61 | Parisi, J. M., Roberts, L., Szanton, S. L., & Hodgson, N. A. (2017). Valued Activities among Individuals with and without Cognitive Impairments: Findings from the National Health and Aging Trends Study. Gerontologist, 57(2), 309–318.  <https://doi.org/10.1093/geront/gnv144> |
| 62 | Parisi, J. M., Roberts, L., Szanton, S. L., Hodgson, N. A., & Gitlin, L. N. (2019). Valued Activities Among Individuals With and Without Functional Impairments: Findings from the National Health and Aging Trends study (NHATS). Activities, Adaptation and Aging, 43(4), 259–275. <https://doi.org/10.1080/01924788.2018.1521254> |
| 63 | Payyanadan, R. P., & Lee, J. D. (2018). Understanding the ridesharing needs of older adults. Travel Behaviour and Society, 13, 155–164.  <https://doi.org/10.1016/j.tbs.2018.08.002> |
| 64 | Payyanadan, R. P., Lee, J. D., & Grepo, L. C. (2018). Challenges for older drivers in Urban, Suburban, and rural settings. Geriatrics (Switzerland), 3(2).  <https://doi.org/10.3390/geriatrics3020014> |
| 65 | Peacock, A., & Pemberton, S. (2019). The paradox of mobility for older people in the rural-urban fringe. Journal of Rural Studies, 70, 9–18.  <https://doi.org/10.1016/j.jrurstud.2019.08.002> |
| 66 | Peterson, J. R., Baumgartner, D. A., & Austin, S. L. (2020). Healthy ageing in the far North: perspectives and prescriptions. International Journal of Circumpolar Health, 79(1). <https://doi.org/10.1080/22423982.2020.1735036> |
| 67 | Plastow, N. A. (2017). Reconsidering the “asphalt Identikit”: A Qualitative Analysis of Driving Identities in British Older Adults in West London. International Journal of Aging and Human Development, 84(4), 403–414.  <https://doi.org/10.1177/0091415016677970> |
| 68 | Pristavec, T. (2018). Social Participation in Later Years: The Role of Driving Mobility. Journals of Gerontology - Series B Psychological Sciences and Social Sciences, 73(8), 1457–1469. <https://doi.org/10.1093/geronb/gbw057> |
| 69 | Rahman, M. M., Deb, S., Strawderman, L., Smith, B., & Burch, R. (2019). Evaluation of transportation alternatives for aging population in the era of self-driving vehicles. IATSS Research. <https://doi.org/10.1016/j.iatssr.2019.05.004> |
| 70 | Rahman, M. M., Strawderman, L., Adams-Price, C., & Turner, J. J. (2016). Transportation alternative preferences of the aging population. Travel Behaviour and Society, 4, 22–28. <https://doi.org/10.1016/j.tbs.2015.12.003> |
| 71 | Ramachandran, M., & D’Souza, S. A. (2016). A cross-sectional survey on older adults’ community mobility in an Indian metropolis. Journal of Cross-Cultural Gerontology, 31(1), 19–33. <https://doi.org/10.1007/s10823-015-9276-7> |
| 72 | Ramachandran, M., & Dsouza, S. A. (2018). Older Adults’ Experiences of Community Mobility in an Indian Metropolis: A Qualitative Study. Physical and Occupational Therapy in Geriatrics, 36(2–3), 315–329. <https://doi.org/10.1080/02703181.2018.1508170> |
| 73 | Ren, G., Zhang, T., Xu, L., & Yang, Y. (2018). Transportation demands of low-mobility individuals: Case study in Wenling, China. Journal of Urban Planning and Development, 144. <https://doi.org/10.1061/(ASCE)UP.1943-5444.0000487> |
| 74 | Ruggiano, N., Shtompel, N., Whiteman, K., & Sias, K. (2017). Influences of Transportation on Health Decision-Making and Self-Management Behaviors among Older Adults with Chronic Conditions. Behavioral Medicine, 43(1), 61–70.  <https://doi.org/10.1080/08964289.2015.1065788> |
| 75 | Ryan, J., Svensson, H., Rosenkvist, J., Schmidt, S. M., & Wretstrand, A. (2016). Cycling and cycling cessation in later life: Findings from the city of Malmö. Journal of Transport and Health, 3(1), 38–47. <https://doi.org/10.1016/j.jth.2016.01.002> |
| 76 | Ryan, Jean, & Wretstrand, A. (2019). What’s mode got to do with it? Exploring the links between public transport and car access and opportunities for everyday activities among older people. Travel Behaviour and Society, 14, 107–118.  <https://doi.org/10.1016/j.tbs.2018.10.003> |
| 77 | Ryan, Jean, Wretstrand, A., & Schmidt, S. M. (2019). Disparities in mobility among older people: Findings from a capability-based travel survey. Transport Policy, 79, 177–192. <https://doi.org/10.1016/j.tranpol.2019.04.016> |
| 78 | Sasaki, K., Aihara, Y., & Yamasaki, K. (2017). The effect of accessibility on aged people’s use of long-term care service. Transportation Research Procedia, 25, 4381–4391. <https://doi.org/10.1016/j.trpro.2017.05.320> |
| 79 | Schatz, E., Seeley, J., Negin, J., & Mugisha, J. (2018). They “don’t cure old age”: Older Ugandans’ delays to health-care access. Ageing and Society, 38(11), 2197–2217. <https://doi.org/10.1017/S0144686X17000502> |
| 80 | Shergold, I. (2019). Taking part in activities, an exploration of the role of discretionary travel in older people’s wellbeing. Journal of Transport and Health, 12, 195–205. <https://doi.org/10.1016/j.jth.2019.01.005> |
| 81 | Smith, M. L., Prohaska, T. R., MacLeod, K. E., Ory, M. G., Eisenstein, A. R., Ragland, D. R., Irmiter, C., Towne Jr., S. D., & Satariano, W. A. (2017). Non-Emergency Medical Transportation Needs of Middle-Aged and Older Adults: A Rural-Urban Comparison in Delaware, USA. International Journal of Environmental Research and Public Health, 14(2 PG-).  <https://doi.org/10.3390/ijerph14020174> |
| 82 | Soltani, A., Pojani, D., Askari, S., & Masoumi, H. E. (2018). Socio-demographic and built environment determinants of car use among older adults in Iran. Journal of Transport Geography, 68, 109–117. <https://doi.org/10.1016/j.jtrangeo.2018.03.001> |
| 83 | Srichuae, S., Nitivattananon, V., & Perera, R. (2016). Aging society in Bangkok and the factors affecting mobility of elderly in urban public spaces and transportation facilities. IATSS Research, 40(1), 26–34. <https://doi.org/10.1016/j.iatssr.2015.12.004> |
| 84 | Stancliffe, R. J., Nye-Lengerman, K. M., & Kramme, J. E. D. (2019). Aging, Community-Based Employment, Mobility Impairment, and Retirement: National Core Indicators–Adult Consumer Survey Data. Research and Practice for Persons with Severe Disabilities, 44(4), 251–266. <https://doi.org/10.1177/1540796919882921> |
| 85 | Stjernborg, V. (2017). The Meaning of Social Participation for Daily Mobility in Later Life: an Ethnographic Case Study of a Senior Project in a Swedish Urban Neighbourhood. Ageing International, 42(3), 374–391. <https://doi.org/10.1007/s12126-017-9296-4> |
| 86 | Strohmeier, F. (2016). Barriers and their influence on the mobility behavior of elder pedestrians in urban areas: challenges and best practice for walkability in the city of Vienna. TRANSPORT RESEARCH ARENA TRA2016, 14(PG-1134-1143), 1134–1143.  <https://doi.org/10.1016/j.trpro.2016.05.184> |
| 87 | Sun, Y., Phillips, D. R., & Wong, M. (2018). A study of housing typology and perceived age-friendliness in an established Hong Kong new town: A person-environment perspective. Geoforum, 88, 17–27. <https://doi.org/10.1016/j.geoforum.2017.11.001> |
| 88 | Talmage, C. A., Knopf, R. C., Wu, T., Winkel, D., Mirchandani, P., & Candan, K. S. (2020). Decreasing Loneliness and Social Disconnectedness among Community-Dwelling Older Adults: The Potential of Information and Communication Technologies and Ride-Hailing Services. Activities, Adaptation and Aging.  <https://doi.org/10.1080/01924788.2020.1724584> |
| 89 | Turner, J. J., Adams-Price, C. E., & Strawderman, L. (2017). Formal Alternative Transportation Options for Older Adults: An Assessment of Need. Journal of Gerontological Social Work, 60(8), 619–646. <https://doi.org/10.1080/01634372.2017.1375590> |
| 90 | Van Cauwenberg, J., Clarys, P., De Bourdeaudhuij, I., Ghekiere, A., de Geus, B., Owen, N., & Deforche, B. (2018). Environmental influences on older adults’ transportation cycling experiences: A study using bike-along interviews. Landscape and Urban Planning, 169(PG-37-46), 37–46. <https://doi.org/10.1016/j.landurbplan.2017.08.003> |
| 91 | Van Cauwenberg, J., De Bourdeaudhuij, I., Clarys, P., De Geus, B., & Deforche, B. (2019). Older adults’ environmental preferences for transportation cycling. Journal of Transport and Health, 13, 185–199. <https://doi.org/10.1016/j.jth.2019.03.014> |
| 92 | Van Cauwenberg, J., De Bourdeaudhuij, I., Clarys, P., Nasar, J., Salmon, J., Goubert, L., & Deforche, B. (2016). Street characteristics preferred for transportation walking among older adults: A choice-based conjoint analysis with manipulated photographs. International Journal of Behavioral Nutrition and Physical Activity, 13(1), 1–17.  <https://doi.org/10.1186/s12966-016-0331-8> |
| 93 | van Hoven, B., & Meijering, L. (2019). Mundane mobilities in later life - Exploring experiences of everyday trip-making by older adults in a Dutch urban neighbourhood. Research in Transportation Business and Management, 30(August), 100375.  <https://doi.org/10.1016/j.rtbm.2019.100375> |
| 94 | Vivoda, J. M., Harmon, A. C., Babulal, G. M., & Zikmund-Fisher, B. J. (2018). E-hail (rideshare) knowledge, use, reliance, and future expectations among older adults. Transportation Research Part F: Traffic Psychology and Behaviour, 55, 426-434  <http://dx.doi.org/10.1016/j.trf.2018.03.020> |
| 95 | Walsh, K., O’Shea, E., & Scharf, T. (2019). Rural old-age social exclusion: A conceptual framework on mediators of exclusion across the lifecourse. Ageing and Society. <https://doi.org/10.1017/S0144686X19000606> |
| 96 | Wong, R. C. P., Szeto, W. Y., Yang, L., Li, Y. C., & Wong, S. C. (2017). Elderly users’ level of satisfaction with public transport services in a high-density and transit-oriented city. Journal of Transport and Health, 7, 209–217.  <https://doi.org/10.1016/j.jth.2017.10.004> |
| 97 | Wormald, A. D., McCallion, P., & McCarron, M. (2019). The antecedents of loneliness in older people with an intellectual disability. Research in Developmental Disabilities, 85, 116–130. <https://doi.org/10.1016/j.ridd.2018.11.009> |
| 98 | Xie, B., An, Z., Zheng, Y., & Li, Z. (2018). Healthy aging with parks: Association between park accessibility and the health status of older adults in urban China. Sustainable Cities and Society, 43, 476–486. <https://doi.org/10.1016/j.scs.2018.09.010> |
| 99 | Yamamoto, F. J., & Zhang, J. (2017). The kindness of strangers: Exploring interdependencies and shared mobilities of elderly people in rural Japan. Social Inclusion, 5(4), 183–195. <https://doi.org/10.17645/si.v5i4.1125> |
| 100 | Zhang, Y., Wu, W., He, Q., & Li, C. (2018). Public transport use among the urban and rural elderly in China: Effects of personal, attitudinal, household, social-environment, and built-environment factors. Journal of Transport and Land Use, 11(1), 701–719.  <https://doi.org/10.5198/jtlu.2018.978> |
